# Increased alcohol use, heavy episodic drinking, and suicide ideation during the COVID-19 pandemic in Canada

**DOI:** 10.17269/s41997-022-00689-7

**Published:** 2022-10-06

**Authors:** Melanie Varin, Li Liu, Robert Gabrys, Geneviève Gariepy, Kate Hill MacEachern, Murray Weeks

**Affiliations:** 1 Public Health Agency of Canada, 785 Carling Avenue, Ottawa, ON K1S 5H4 Canada; 2 Canadian Centre on Substance Use and Addiction, Ottawa, ON Canada; 3 Public Health Agency of Canada, Montreal, QC Canada; 4 Institut Universitaire en Santé Mentale de Montréal, Research Centre, Montreal, QC Canada

**Keywords:** Suicide ideation, Heavy episodic drinking, Increased alcohol use, COVID-19, Canada, Idées suicidaires, consommation épisodique excessive d’alcool, consommation accrue d’alcool, COVID-19, Canada

## Abstract

**Objective:**

Alcohol use is a known risk factor for suicidality, yet this relationship has not been explored during the pandemic in Canada. As a growing body of evidence demonstrates the negative impact of COVID-19 on alcohol consumption and associated harms in Canada, there is a need to examine this more closely.

**Methods:**

Using the Survey on COVID-19 and Mental Health 2020, we compared the prevalence of suicide ideation among: (1) individuals who reported an increase in alcohol consumption vs those who reported a decrease/no change, and (2) individuals who reported past month heavy episodic drinking vs those who did not. We compared overall unadjusted odds ratios and across a number of sociodemographic and mental health variables. All estimates were weighted to ensure they were nationally representative.

**Results:**

The prevalence and likelihood of suicide ideation were significantly higher among people who reported increased alcohol consumption during the pandemic (4.9% vs 2.0%; OR = 2.6, 95% CI: 1.8, 3.7) and people who reported past month heavy episodic drinking (3.4% vs 2.1%; OR = 1.7, 95% CI: 1.2, 2.3). Males and middle-aged and older-aged individuals had the highest odds ratios for increased alcohol consumption and past month heavy episodic drinking with suicide ideation.

**Conclusion:**

In the Canadian general population during the COVID-19 pandemic, there were significant associations between suicide ideation and increased alcohol use as well as past month heavy episodic drinking across specific sociodemographic subgroups. Future research could explore these associations while adjusting for social determinants of health such as income security, employment, education, social support, stress, and mental health.

## Résumé

**Contexte:**

La consommation d’alcool est un facteur de risque connu de suicidabilité, mais cette relation n’a pas été explorée pendant la pandémie au Canada. Comme un nombre croissant de preuves démontre les répercussions négatives de la COVID-19 sur la consommation d’alcool et les méfaits associés au Canada, il est nécessaire d’examiner cette question de plus près.

**Méthodes:**

En utilisant l’enquête de 2020 sur la COVID-19 et la santé mentale, nous avons comparé la prévalence des idées suicidaires chez : (1) les individus qui ont déclaré une augmentation de leur consommation d’alcool par rapport à ceux qui ont déclaré une diminution ou aucun changement, et (2) les individus qui ont déclaré une consommation épisodique excessive d’alcool au cours du dernier mois par rapport à ceux qui n’en ont pas déclaré. Nous avons comparé les rapports des cotes globaux non ajustés et selon un certain nombre de variables sociodémographiques et de santé mentale. Toutes les estimations ont été pondérées afin de garantir leur représentativité nationale.

**Résultats:**

La prévalence et la probabilité d’idées suicidaires étaient considérablement plus élevées chez les personnes ayant déclaré une consommation d’alcool accrue pendant la pandémie (4,9 % contre 2,0 %; RC=2,6, IC à 95 % : 1,8 à 3,7) et chez les personnes ayant déclaré une consommation épisodique excessive au cours du mois écoulé (3,4 % contre 2,1 %; RC=1,7, IC à 95 % : 1,2 à 2,3). Les hommes, les personnes d’âge moyen et les personnes âgées présentaient les rapports des cotes les plus élevés pour une consommation d’alcool accrue et une consommation épisodique excessive d’alcool au cours du mois précédent avec des idées suicidaires.

**Conclusion:**

Dans la population générale canadienne, pendant la pandémie de COVID-19, il y avait des associations significatives entre les idées suicidaires et l’augmentation de la consommation d’alcool ainsi que la consommation épisodique excessive d’alcool au cours du dernier mois dans des sous-groupes sociodémographiques précis. Les recherches futures pourraient explorer ces associations tout en ajustant les déterminants sociaux de la santé tels que la sécurité du revenu, l’emploi, l’éducation, le soutien social, le stress et la santé mentale.

## Introduction

Since the declaration of the COVID-19 pandemic in early 2020, its wider consequences, including the potential impacts on population mental health and well-being, have been examined (Canadian Centre on Substance Use and Addiction, 2020; Hill MacEachern et al., 2021; Shields et al., 2021). A particular concern raised by researchers has been the risk of suicide during the COVID-19 pandemic (Aquila et al., 2020; Gunnell et al., 2020; John et al., 2020a; McIntyre & Lee, 2020). Evidence depicting increased suicide mortality during and after previous viral epidemics provides support for these concerns (Gunnell et al., 2020; Leaune et al., 2020).

While some studies point towards an increase in suicidality (ideation, attempts, deaths) during the pandemic (Dubé et al., 2021; Tanaka & Okamoto, 2021), others, including a nationally representative Canadian study (Liu et al., 2021), show no change (Appleby, 2021; Ayuso-Mateos et al., 2021; Faust et al., 2021; John et al., 2020a). These different results are not surprising as there are many contextual factors that could influence suicidality during COVID-19 including variations in types of lockdowns, country responses, policies, and rates of infections. In addition, certain suicide-related risk factors such as social isolation, loneliness, fear of contracting or spreading the disease, interpersonal violence, access to means, and alcohol misuse have been exacerbated during the COVID-19 pandemic (Aquila et al., 2020; Gunnell et al., 2020; McIntyre & Lee, 2020). Financial stressors have also intensified during this time (Aquila et al., 2020; Elbogen et al., 2021; Gunnell et al., 2020; McIntyre & Lee, 2020), which is concerning as previous evidence demonstrated an increase in the percentage of suicide deaths with an acute alcohol intoxication after the 2008 economic recession (Chang et al., 2013; Kaplan et al., 2015). The magnitude was particularly high in countries where unemployment was low before the economic crisis (Chang et al., 2013). Additional information on alcohol and suicide during the COVID-19 pandemic in Canada is needed.

The relationship between alcohol use and suicide is complex and involves different mechanisms (Mental Health Commission of Canada, 2022). Acute use of alcohol can increase the risk of suicide by impairing judgement, increasing impulsivity, and reducing inhibition, whereas heavy drinking can increase the risk through difficulties in regulating emotions, onset of major depression, or stressful life events (Mental Health Commission of Canada, 2022). There is a growing body of evidence demonstrating the negative impact of COVID-19 on alcohol consumption (Canadian Centre on Substance Use and Addiction, 2020; Hill MacEachern et al., 2021) and associated harms among Canadians (Canadian Institute for Health Information, 2021). This is concerning as alcohol use is a known risk factor for suicidality, and individuals with alcohol dependence have an estimated 10-fold increase in suicide risk (pooled relative risk: 9.8, 95% CI: 9.0, 10.7) (Ferrari et al., 2014).

A few international studies have demonstrated that alcohol use was significantly associated with increased odds of suicide ideation during the pandemic (Bonsaksen et al., 2021; Elbogen et al., 2021; Mamun et al., 2021). To date, no Canadian study has assessed this relationship.

Accordingly, the goal of the present study was to address this gap by examining the relationship between alcohol use and suicide ideation in a study of a large nationally representative sample conducted during the second wave of the COVID-19 pandemic (September to December 2020). The aims of this study were to:

1. Compare the prevalence of suicide ideation in adults (aged 18+) reporting change in alcohol consumption (increased vs decreased/no change) and heavy episodic drinking (yes vs no) in Canada.
2. Examine the relationship between change in alcohol use and suicide ideation across sociodemographic characteristics and mental health conditions.
3. Examine the relationship between heavy episodic drinking and suicide ideation across sociodemographic characteristics and mental health conditions.

## Methods

### Survey design

The Survey on COVID-19 and Mental Health (SCMH) is a nationally representative cross-sectional survey of people living in Canada led by Statistics Canada and the Public Health Agency of Canada (PHAC) conducted from September 11 to December 4, 2020. The survey sampling frame was stratified by province and a simple random sample of 30,000 dwellings was selected within each province and within the three territorial capital cities. Respondents were sent a letter through mail with information on how to complete the survey electronically on the Statistics Canada website. Statistics Canada interviewers subsequently contacted individuals who had not responded to the electronic questionnaire to complete the survey by computer-assisted telephone interview. A total of 14,689 individuals aged 18 years and older completed the survey, representing a response rate of 53.3%. Of this sample, 84% agreed to share their data with PHAC (*n* = 12,344). The survey coverage excluded individuals living in institutions, individuals living on reserves or other Indigenous settlements, and full-time members of the Canadian Armed Forces. Overall, these exclusions are estimated to be less than 2% of the population. This study was based on de-identified secondary data shared with PHAC under the authority of the Federal Statistics Act (Government of Canada, 1985). Participation in this survey was voluntary and respondents gave their informed consent to take part in the study. PHAC signed a data sharing agreement with Statistics Canada to get access to information provided in the survey. Confidentiality of all respondents was maintained and there was no identifiable information in the shared data file. Therefore, research ethics board approval was not required. Additional information on the survey methodology can be found on Statistics Canada’s website (Statistics Canada, 2021).

### Measures

*Suicide ideation during the COVID-19 pandemic* was measured with the question: “Have you seriously contemplated suicide since the COVID-19 pandemic began?” Response options included “yes” or “no”.

*Change in alcohol use* was assessed by the following item: “On average, over the course of the COVID-19 pandemic, how has your alcohol consumption changed when comparing to before the pandemic?” Response options included “increased”, “decreased”, and “no change”. For this study, “decreased” and “no change” response options were combined, creating a dichotomous variable: (i) increased vs (ii) decreased/no change.

*Heavy episodic drinking* refers to males who reported having five or more drinks or females who reported having four or more drinks on one occasion during the past 30 days (Canadian Centre on Substance Use and Addiction, 2018). In the SCMH, heavy episodic drinking was assessed by asking respondents the following question: “During the past 30 days, how often have you had 4+/5+ drinks on one occasion?”, with the following response options: “daily or almost daily”, “2 to 5 times a week”, “once a week”, “2 to 3 times in the past 30 days”, “once in the past 30 days” and “not in the past 30 days”. Respondents who selected any response option other than “not in the past 30 days” were classified as part of the “heavy episodic drinking” group. By contrast, respondents who selected “not in the past 30 days” OR “did not drink alcohol in the past 30 days” were classified as part of the “non-heavy episodic drinking” group. Heavy episodic drinking is considered a behaviour that exceeds Canada’s Low-Risk Alcohol Drinking Guidelines (Canadian Centre on Substance Use and Addiction, 2018) and can lead to harm.

#### Mental health variables

Respondents who had a score of 10 or more (out of 21) on the 7-item generalized anxiety disorder (GAD) scale (GAD-7) (Spitzer et al., 2006) and a score of 10 or more (out of 27) on the 9-item Patient Health Questionnaire (PHQ-9) (Kroenke et al., 2001) were considered to have moderate to severe symptoms of GAD and major depressive disorder (MDD), respectively, in the past 2 weeks. Henceforth, we refer to these respondents as those who screened positive for GAD and/or MDD. The 20-item posttraumatic stress disorder (PTSD) Checklist for DSM-5 (PCL-5) assesses posttraumatic stress symptoms in the past month such as repeated/disturbing/unwanted memories, avoiding external reminders, and being super alert/on guard. Respondents with a score of 33 or more (out of 80) were considered to meet the cut-point for probable PTSD (Blevins et al., 2015). It should be noted that the event causing the PTSD is not specified and could include probable PTSD due to COVID-19 or other events that occurred in the past month.

#### Sociodemographic variables

Education (high school and lower; post-secondary education), age group (18 to 34 years; 35 to 64 years; 65+), total household income level (low; middle; high), being a parent or legal guardian of a child or children under the age of 18 (yes; no), living area (urban; rural), immigrant status (yes, which includes individuals who are or who have ever been landed immigrants or permanents residents, as well as non-permanent residents; no, which includes individuals born in Canada), and people who self-identify as being part of a racialized group (yes; no) were included as sociodemographic variables. Gender was measured with the question, “What is your gender? Gender refers to current gender which may be different from sex assigned at birth and may be different from what is indicated on legal documents. Is it: “Male”, “Female”, or “please specify your gender.” As this was a secondary data analysis limited by the survey question and responses, for rigour and ethic purposes it was chosen to report on gender using the answer choices that were provided (male, female) to the people surveyed. Racialized group membership was measured with the visible minority question asking respondents to identify the population group or groups to which they belong. The Employment Equity Act defines visible minorities as “people other than Indigenous who are non-Caucasian in race or non-white in colour”. Not stated responses were deemed as missing. Due to the small number of observations, we did not include the gender diverse category in the gender variable, but these participants were included in all other analyses.

### Statistical analyses

We compared the prevalence of suicide ideation since the pandemic began in two pairs of groups:

1. Individuals who reported increased alcohol consumption since the pandemic began compared to those who reported a decrease or no change; and
2. Individuals who reported past month heavy episodic drinking compared to those who did not.

We calculated weighted proportions and 95% confidence intervals (CI). Due to the low prevalence of suicide ideation, the sampling distribution did not follow a normal distribution. We therefore reported the Clopper-Pearson 95% CIs (Clopper & Pearson, 1934). All estimates were disaggregated by sociodemographic and mental health variables.

We then performed unadjusted logistic regression analyses to estimate the odds ratios of suicide ideation in the two groups and across each sociodemographic and mental health variable. All analyses were weighted to be representative of the Canadian adult population. To account for the complex design of the survey, replicate weights and the bootstrapping method were used for variance estimation. We used SAS Enterprise Guide version 7.1 for statistical analyses.

## Results

Overall, 2.4% of Canadian adults reported having seriously contemplated suicide since the beginning of the pandemic. In addition, 15.7% of Canadian adults reported increased alcohol use, and 28.8% reported past month heavy episodic drinking. Compared to individuals with no suicide ideation since the beginning of COVID-19, those who reported suicide ideation had much higher prevalence of probable PTSD (50.2% vs 5.2%), as well as screening positive for MDD (66.4% vs 11.8%) and/or GAD (73.5% vs 13.7%). Increased alcohol use during the pandemic (31.8% vs 15.4%) and heavy episodic drinking (40.0% vs 28.6%) were also more common among individuals who reported suicide ideation relative to those who did not. Prevalence of suicide ideation varied significantly across most sociodemographic groups except for gender, racialized group membership, and parents/legal guardian status (see Table 1).

**Table 1:** Description of the survey population

Variable	Suicide ideation (*n* = 316) % (95% CI)	No suicide ideation (*n* = 11,976) % (95% CI)	Chi-square test *p* value
Overall	2.4 (2.0, 2.9)	97.6 (97.2, 98.0)	0.162
Gender			
Male	42.9 (34.1, 51.6)	49.3 (49.1, 49.6)	0.162
Female	57.1 (48.4, 65.9)	50.7 (50.4, 50.9)	
Age group			
18 to 34 years	48.9 (40.3, 57.5)	27.6 (27.3, 27.9)	<0.001
35 to 64 years	46.2 (37.9, 54.5)	49.7 (49.5, 50.0)	
65+ years	4.9 (2.4, 7.5)	22.7 (22.5, 22.8)	
Racialized group member			
Yes	14.1 (6.2, 22.1)	24.4 (23.2, 25.6)	0.046
No	85.9 (77.9, 93.9)	75.6 (74.4, 76.8)	
Immigrant status			
Yes	15.1 (7.3, 23.0)	27.2 (26.0, 28.4)	0.018
No	84.9 (77.0, 92.7)	72.8 (71.6, 74.0)	
Total household income level			
Low	48.8 (40.2, 57.4)	34.8 (33.5, 36.1)	0.001
Middle	31.0 (23.1, 38.8)	31.8 (30.5, 33.2)	
High	20.3 (13.3, 27.3)	33.4 (32.1, 34.8)	
Living area			
Urban	87.8 (83.0, 92.7)	82.2 (81.3, 83.0)	0.050
Rural	12.2 (7.4, 17.0)	17.9 (17.0, 18.7)	
Education level			
High school graduate or less	42.2 (33.6, 50.7)	31.0 (29.7, 32.2)	0.007
Post-secondary graduate	57.8 (49.3, 66.4)	69.0 (67.8, 70.3)	
Parent or legal guardian			
Yes	25.9 (18.8, 33.0)	27.6 (26.7, 28.6)	0.644
No	74.1 (67.0, 81.2)	72.4 (71.4, 73.3)	
Screened positive for GAD			
Yes	73.5 (65.0, 82.1)	13.7 (12.7, 14.7)	<0.001
No	26.5 (17.9, 35.0)	86.3 (85.3, 87.3)	
Screened positive for MDD			
Yes	66.4 (58.7, 74.1)	11.8 (10.9, 12.8)	<0.001
No	33.6 (25.9, 41.3)	88.2 (87.3, 89.1)	
Screened positive for PTSD			
Yes	50.2 (41.3, 59.2)	5.2 (4.6, 5.9)	<0.001
No	49.8 (40.8, 58.8)	94.8 (94.1, 95.4)	
Change in alcohol consumption			
Increased	31.8 (24.1, 39.5)	15.4 (14.4, 16.3)	<0.001
Decreased/no change	68.2 (60.5, 75.9)	84.6 (83.7, 85.6)	
Past month heavy episodic drinking			
Yes	40.0 (32.2, 47.8)	28.6 (27.4, 29.8)	0.002
No	60.0 (52.3, 67.8)	71.4 (70.2, 72.6)	

Data source: Survey on COVID-19 and Mental Health, September–December 2020

*GAD* generalized anxiety disorder, *MDD* major depressive disorder, *PTSD* posttraumatic stress disorder

### Prevalence estimates of suicide ideation for change in alcohol consumption and heavy episodic drinking

Among those who reported increased alcohol use and past month heavy episodic drinking, the highest absolute prevalence estimates of suicide ideation were among people with probable PTSD (22.2% and 18.6%), those who screened positive for GAD (13.8% and 14.9%), those who screened positive for MDD (14.4% and 13.6%), individuals in the 18–34 age group (6.2% and 4.8%), those who had a high school education or lower (8.7% and 4.6%), or those from the lowest income level (8.3% and 5.3%).

### Change in alcohol consumption and suicide ideation

Table 2 presents the overall and disaggregated prevalence estimates and odds ratios for suicide ideation across changes in alcohol consumption. The prevalence and likelihood of suicide ideation were significantly higher among people who reported increased alcohol consumption compared to those who reported a decrease or no change (4.9% vs 2.0%; OR = 2.6, 95% CI: 1.8, 3.7). After disaggregation, we found that this association was also significant among males, females, individuals aged 35+, non-racialized group members, non-immigrants, all total household income levels, urban and rural areas, and all education levels. Of these groups, the odds ratios were highest among males (OR = 3.6, 95% CI: 2.0, 6.7), those 65 years and older (OR = 5.5, 95% CI: 1.6, 18.6), and those with a high school education or lower (OR = 3.4, 95% CI: 1.7, 6.9). There were no significant associations between increased alcohol consumption and suicide ideation for the 18 to 34 age group, racialized group members, immigrants, those with probable PTSD, those who screened positive for GAD, and those who screened positive for MDD.

**Table 2:** Prevalence estimates and odds ratios of suicide ideation among adults who self-reported alcohol consumption increased vs decreased/no change, and disaggregated by sociodemographic characteristics and mental health conditions

Variable	Increased (*n* = 1893) % (95% CI)	Decreased/no change (*n* = 10,364) % (95% CI)	Chi-square test *p* value	Odds ratio Increased vs decreased/no change
Overall	4.9 (3.6, 6.6)	2.0 (1.6, 2.4)	<0.001	2.6 (1.8, 3.7)
Gender				
Male	5.3 (3.3, 8.0)	1.5 (1.0, 2.2)	<0.001	3.6 (2.0, 6.7)
Female	4.5 (2.9, 6.6)	2.3 (1.8, 2.9)	0.003	2.0 (1.2, 3.2)
Age group				
18 to 34 years	6.2 (3.3, 10.5)	3.8 (2.7, 5.3)	0.132	1.7 (0.8, 3.3)
35 to 64 years	4.7 (3.3, 6.6)	1.7 (1.3, 2.2)	<0.001	2.9 (1.9, 4.5)
65+ years	2.2 (0.7, 5.1)	0.4 (0.2, 0.8)	<0.001	5.5 (1.6, 18.6)
Racialized group member				
Yes	F	1.4 (0.6, 2.8)	0.700	1.3 (0.1, 13.1)
No	5.4 (3.9, 7.3)	2.1 (1.7, 2.6)	<0.001	2.6 (1.8, 3.8)
Immigrant status				
Yes	2.0 (0.5, 5.1)	1.3 (0.6, 2.5)	0.490	1.5 (0.4, 6.1)
No	5.5 (3.9, 7.4)	2.3 (1.8, 2.8)	<0.001	2.5 (1.7, 3.7)
Total household income level				
Low	8.3 (5.3, 12.3)	2.8 (2.1, 3.6)	<0.001	3.1 (1.9, 5.2)
Middle	4.7 (2.2, 8.5)	1.9 (1.3, 2.8)	0.013	2.5 (1.2, 5.4)
High	3.0 (1.6, 5.1)	1.0 (0.5, 1.6)	0.001	3.2 (1.5, 7.0)
Living area				
Urban	5.1 (3.6, 7.0)	2.1 (1.7, 2.7)	<0.001	2.5 (1.6, 3.7)
Rural	3.9 (1.6, 7.9)	1.3 (0.8, 2.2)	0.001	3.0 (1.1, 8.0)
Education level				
High school graduate or less	8.7 (4.5, 14.7)	2.7 (1.9, 3.8)	<0.001	3.4 (1.7, 6.9)
Post-secondary graduate	4.0 (2.8, 5.5)	1.6 (1.2, 2.1)	<0.001	2.6 (1.7, 3.9)
Parent or legal guardian				
Yes	3.9 (2.1, 6.5)	1.8 (1.2, 2.7)	<0.001	2.2 (1.1, 4.5)
No	5.6 (3.8, 8.0)	2.0 (1.6, 2.6)	0.019	2.9 (1.8, 4.5)
Screened positive for GAD				
Yes	13.8 (9.2, 19.6)	11.8 (8.9, 15.1)	0.480	1.2 (0.7, 2.0)
No	2.2 (1.3, 3.5)	0.7 (0.5, 1.0)	<0.001	3.0 (1.7, 5.3)
Screened positive for MDD				
Yes	14.4 (10.2, 19.6)	11.0 (8.7, 13.6)	0.153	1.4 (0.9, 2.1)
No	1.7 (0.8, 3.0)	0.6 (0.4, 1.0)	0.008	2.7 (1.2, 6.0)
Probable PTSD				
Yes	22.2 (14.2, 31.9)	17.9 (13.5, 23.0)	0.367	1.3 (0.7, 2.4)
No	2.6 (1.7, 3.9)	1.0 (0.7, 1.4)	<0.001	2.6 (1.5, 4.5)

Data source: Survey on COVID-19 and Mental Health, September–December 2020

*GAD* generalized anxiety disorder, *MDD* major depressive disorder, *PTSD* posttraumatic stress disorder

^F^As per the release guidelines for the SCMH, estimates are deemed of poor quality due to high variability and should not be released

### Past month heavy episodic drinking and suicide ideation

Table 3 presents the overall and disaggregated prevalence estimates and odds ratios for suicide ideation across past month heavy episodic drinking. The prevalence and likelihood of suicide ideation were significantly higher among people who reported past month heavy episodic drinking compared to those who did not (3.4% vs 2.1%; OR = 1.7, 95% CI: 1.2, 2.3). The association between past month heavy episodic drinking and suicide ideation was significant for males, females, people aged 35 years and over, non-racialized group members, non-immigrants, individuals in the low or middle household income groups, people living in an urban area, post-secondary education, and individuals without a child or children under the age of 18 in the household. The odds ratios were highest among the 65+ age group (OR = 4.2, 95% CI: 1.3, 13.6) and males (OR = 2.1, 95% CI: 1.2, 3.7). There were no significant associations between past month heavy episodic drinking and suicide ideation for the 18 to 34 age group, racialized group members, immigrants, individuals in the highest income bracket, people living in a rural area, people with a high school education or lower, parents/caregivers, people with probable PTSD, those who screened positive for GAD, and those who screened positive for MDD.

**Table 3:** Prevalence estimates and odds ratio of suicide ideation among adults who self-reported past month heavy episodic drinking (yes vs no), and disaggregated by sociodemographic characteristics and mental health conditions

Variable	Yes (*n* = 3779) % (95% CI)	No (*n* = 8467) % (95% CI)	Chi-square test *p* value	Odds ratio Yes vs no
Overall	3.4 (2.6, 4.3)	2.1 (1.6, 2.6)	0.002	1.7 (1.2, 2.3)
Gender				
Male	3.2 (2.2, 4.7)	1.6 (1.0, 2.4)	0.008	2.1 (1.2, 3.7)
Female	3.6 (2.5, 4.8)	2.4 (1.8, 3.1)	0.046	1.5 (1.0, 2.3)
Age group				
18 to 34 years	4.8 (3.1, 7.1)	4.0 (2.6, 5.7)	0.495	1.2 (0.7, 2.1)
35 to 64 years	3.1 (2.2, 4.2)	1.9 (1.4, 2.5)	0.024	1.6 (1.1, 2.5)
65+ years	1.4 (0.6, 2.9)	0.3 (0.1, 0.7)	0.003	4.2 (1.3, 13.6)
Racialized group member				
Yes	1.6 (0.4, 3.9)	1.4 (0.6, 2.8)	0.832	1.1 (0.3, 5.2)
No	3.6 (2.8, 4.7)	2.3 (1.8, 2.9)	0.007	1.6 (1.1, 2.3)
Immigrant status				
Yes	2.0 (0.8, 4.1)	1.3 (0.5, 2.6)	0.360	1.6 (0.5, 4.9)
No	3.7 (2.8, 4.7)	2.5 (1.9, 3.1)	0.021	1.5 (1.1, 2.2)
Total household income level				
Low	5.3 (3.7, 7.4)	2.8 (2.0, 3.7)	0.003	2.0 (1.3, 3.2)
Middle	3.6 (2.1, 5.7)	1.8 (1.1, 2.7)	0.026	2.1 (1.1, 4.0)
High	1.9 (1.0, 3.4)	1.2 (0.7,1.9)	0.203	1.6 (0.7, 3.6)
Living area				
Urban	3.6 (2.7, 4.7)	2.2 (1.7, 2.8)	0.007	1.6 (1.1, 2.4)
Rural	2.4 (1.3, 4.2)	1.4 (0.7, 2.3)	0.125	1.8 (0.8, 4.2)
Education level				
High school graduate or less	4.6 (2.9, 6.9)	2.8 (1.8, 4.2)	0.094	1.7 (0.9, 3.0)
Post-secondary graduate	2.9 (2.1, 3.9)	1.7 (1.3, 2.2)	0.010	1.7 (1.1, 2.6)
Parent or legal guardian				
Yes	3.0 (1.7, 4.8)	2.0 (1.3, 3.0)	0.231	1.5 (0.7, 3.0)
No	3.6 (2.7, 4.7)	2.1 (1.6, 2.7)	0.004	1.7 (1.2, 2.6)
Screened positive for GAD				
Yes	14.9 (11.1, 19.5)	10.9 (7.9, 14.6)	0.127	1.4 (0.9, 2.3)
No	1.1 (0.6, 1.7)	0.9 (0.6,1.2)	0.579	1.2 (0.7, 2.1)
Screened positive for MDD				
Yes	13.6 (10.2, 17.6)	10.9 (8.5, 13.9)	0.220	1.3 (0.9, 1.9)
No	0.9 (0.5, 1.5)	0.7 (0.4,1.2)	0.639	1.2 (0.5, 2.8)
Probable PTSD				
Yes	18.6 (12.6, 26.0)	19.2 (14.2, 25.0)	0.900	1.0 (0.6, 1.7)
No	2.0 (1.4, 2.8)	1.0 (0.7, 1.4)	0.005	2.1 (1.2, 3.4)

Data source: Survey on COVID-19 and Mental Health, September–December 2020

*GAD* generalized anxiety disorder, *MDD* major depressive disorder, *PTSD* posttraumatic stress disorder

## Discussion

Overall, the prevalence and likelihood of suicide ideation were significantly higher among individuals who reported increased alcohol use during the pandemic and past month heavy episodic drinking compared to individuals whose consumption did not change/decreased and those who did not report heavy episodic drinking in the past month. These findings provide additional support to the importance of evidence-based suicide prevention strategies during and in the recovery phase of the COVID-19 pandemic (Wasserman et al., 2020). Screening programs among people who use alcohol in a more harmful way and alcohol policies (availability, marketing, and pricing) aimed at reducing harmful use of alcohol have previously been suggested as selective preventive strategies to consider (Wasserman et al., 2020). Universal strategies such as increasing public health education related to alcohol and suicide, including the ways to reduce the risk of alcohol-related suicide at the individual level among Canadians (Mental Health Commission of Canada, 2022), could also help reduce the risk of suicide (Wasserman et al., 2020).

Three international epidemiological studies that assessed associations between alcohol and suicide ideation during the COVID-19 pandemic support those results (Bonsaksen et al., 2021; Elbogen et al., 2021; Mamun et al., 2021). First, a Norwegian study found that adults who consumed alcohol daily were three times more likely (adjusted OR = 3.3, 95% CI: 1.4, 7.9) to experience past month suicide ideation (Bonsaksen et al., 2021), although it should be noted that the majority of that sample were women (85%). There was also a population-based study of over 10,000 individuals from Bangladesh that found that people who reported using alcohol had close to twice the odds (OR = 1.8, 95% CI: 1.2, 2.8) of suicide ideation than those who did not use alcohol (Mamun et al., 2021). Last, a national US survey of 6607 adults found a significant albeit weak association of suicide ideation (OR = 1.1, 95% CI: 1.0, 1.1) with increased risk for alcohol use disorder (Elbogen et al., 2021). Two considerations should be noted when comparing our findings to these three studies: (1) alcohol measures differed, and (2) not all alcohol use leads to harms. Our study adds weight to this body of evidence by showing a similar association between alcohol use beyond low-risk guidelines and suicide ideation during the pandemic in Canada.

Across sociodemographic characteristics, we found that males and middle-aged and older-aged individuals had the highest odds ratios for increased alcohol consumption and past month heavy episodic drinking with suicide ideation. Interestingly, the prevalence of both alcohol outcomes was lowest among the 65+ age group compared to the other age groups, while the odds ratios had the highest magnitude. Our findings are consistent with previous research identifying harmful alcohol use as a risk factor for suicide among older individuals (Conwell & Duberstein, 2005). Further, the existing literature demonstrates that, compared to females, males are more likely to engage in risky alcohol consumption and are more likely to have thoughts of suicide (Turecki & Brent, 2016).

As for the mental health conditions, the highest prevalence estimates of suicide ideation were among people who screened positive for a mental health disorder (PTSD, GAD, MDD) and who reported increased alcohol use or past month heavy episodic drinking. Approximately one in five individuals with probable PTSD who reported increased alcohol use (22.2%) or past month heavy episodic drinking (18.6%) also reported suicide ideation. A similar pattern was seen among individuals who screened positive for GAD and who screened positive for MDD, but with lower proportions (between 13% and 15%). This may be due to the over-representation of people who screened positive for a mental disorder in our sample of individuals with suicide ideation. Interestingly, no significant differences or associations were found between increased alcohol use and past month heavy episodic drinking with suicide ideation for people with probable PTSD, nor for individuals who screened positive for GAD or MDD. However, we did find that individuals who did not screen positive for a mental disorder (PTSD, GAD, MDD) and who had thoughts of suicide ideation were significantly more likely to report increased alcohol consumption and past month heavy episodic drinking. Mental health conditions are known to be strong risk factors for both alcohol consumption (Bolton et al., 2006) and suicide ideation (Hawton et al., 2013; Krysinska & Lester, 2010), which may have confounded or impacted the relationship between alcohol and suicide ideation.

The present study has some notable strengths. Monitoring risk factors of suicide ideation during the COVID-19 pandemic was highlighted as an important area of action (John et al., 2020b). Our study addressed this need by examining alcohol use, which is a risk factor of suicide ideation during the pandemic. Second, this was the first nationally representative estimate of suicide ideation during COVID-19 among individuals who reported increased alcohol consumption and past month heavy episodic drinking. Third, this is the first population-based study that examined the relationship between increased alcohol consumption, heavy episodic drinking, and suicide ideation across sociodemographic characteristics and mental health conditions during COVID-19.

The interpretation of this study’s findings should also be made with some limitations in mind. As the SCMH is a cross-sectional survey, we were unable to infer temporality and some of the associations may be bi-directional. Due to the nature of the data, we are unable to conclude whether increased alcohol consumption and/or heavy episodic drinking occurred concurrently with suicide ideation. All of the data in the analyses were self-reported and are subject to reporting and social desirability bias. Heavy episodic drinking was defined as any individual who reported consuming 4–5+ drinks at least once in the past 30 days. It should be noted that individuals who reported engaging in this behaviour several times a week or daily may be different than the ones who reported only doing it once in the past 30 days. The analysis did not account for that level of granularity. The number of respondents who reported experiencing suicide ideation during the COVID-19 pandemic was low. This restricted the analysis in the following two ways: (1) unable to disaggregate the data by province and territory to examine whether there are jurisdiction-level differences, and (2) no adjusted logistic regression models could be performed. Some of the significant associations could be explained by other factors. In addition, certain factors such as unemployment, early life trauma, history of suicide ideation, stress, and access to alcohol were not measured, which could result in residual confounding.

## Conclusion

The prevalence of suicide ideation was significantly higher among Canadians who reported that their alcohol consumption had increased since the start of COVID-19 compared to those who reported a decrease/no change in use. The prevalence was also higher among Canadians who reported past month heavy episodic drinking compared to those who reported no past month heavy episodic drinking. Furthermore, increased alcohol use and past month heavy episodic drinking were significantly associated with suicide ideation, particularly among males, middle-aged and older-aged adults, non-immigrants, and people without a mental health condition. It will be important to continue monitoring alcohol use and suicide ideation as the pandemic continues and into the recovery phase, to support development of public education and messaging.

## Contributions to knowledge

What does this study add to existing knowledge?

- There is no information on the relationship between alcohol and suicide ideation in Canada during the COVID-19 pandemic. This study provides the first population-based estimates of alcohol and suicide ideation in Canada.
- These results add to existing knowledge by demonstrating that the prevalence and likelihood of suicide ideation were significantly higher among Canadians who reported that their consumption of alcohol had increased during the pandemic and who reported past month heavy episodic drinking.

What are the key implications for public health interventions, practice, or policy?

- Individuals who consume alcohol may be at increased risk for suicide or suicide-related behaviour.
- Ongoing surveillance of alcohol use and suicide ideation during the COVID-19 pandemic and into the recovery phase would be crucial to support the development of targeted prevention strategies, public education, and messaging about the impacts of alcohol consumption on suicide.
